# An Unusual Case of Bilateral Agenesis of the Cochlear Nerves

**DOI:** 10.1155/2012/581920

**Published:** 2012-07-30

**Authors:** Vítor Yamashiro Rocha Soares, Fabrício Mendes Ferreira, Christiane França Coimbra, André Luiz Lopez Sampaio, Carlos Augusto Costa Pires De Oliveira

**Affiliations:** ^1^Department of Otorhinolaryngology-Head and Neck Surgery, University of Brasilia, DF, Brazil; ^2^Department of Radiology, University of Brasilia, DF, Brazil

## Abstract

Imaging of the cochlea and internal auditory canals are increasingly important nowadays because of the growing number of cochlear implants being performed throughout the world. We report a case of a 4-year-old boy who was born deaf and was being evaluated in our service for possible cochlear implantation. Audiometry disclosed profound bilateral deafness. The magnetic resonance imaging revealed only two nerves in each inner auditory canal: one in the anterior superior quadrant, identified as the facial nerve, and one on the posterior quadrants, representing both the superior and inferior vestibular nerves. The semicircular canals were not seen and the vestibule had dysplastic morphology. The diagnosis was bilateral agenesis of the cochlear nerves and semicircular canals.

## 1. Introduction

Congenital sensorineural hearing loss (SNHL) has an incidence of approximately 1–3 cases per 1.000 live births [1]. In some of these patients, cochlear implantation is a well-established and safe procedure. For this, the presence of the spiral ganglion cells and the cochlear nerve (CN) is required for the signal transmission from a cochlear implant to the brain. Thereby, the preoperative determination of cochlear anatomy is important because cochlear implantation is contraindicated in patients with cochlear nerve aplasia, hypoplasia, or agenesis and inner ear malformations [2].

Imaging of the cochlea and internal auditory canal (IAC) is essential for a cochlear implant candidate in order to establish cochlear duct permeability and the presence of cochlear nerve in the IAC as well as for choosing the side to be implanted [3]. There are few published studies about radiologic images of patients with sensorineural hearing loss in the literature [2, 3]. The joint use of the computerized tomography (CT) and magnetic resonance imaging (MRI) supplies information on the internal auditory canal thickness, presence of the cochlear, vestibular, and facial nerves, and inner ear malformations [3].

**Figure 1:**
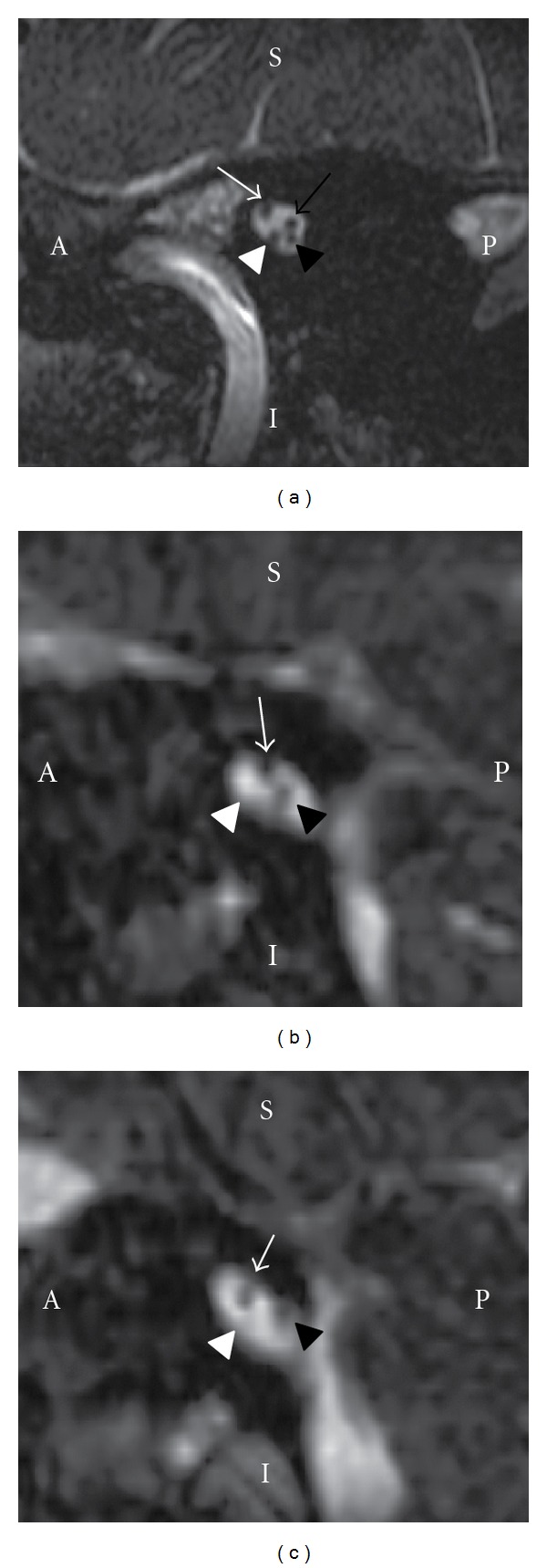
(a) Normal sagittal oblique 3D CISS MR Image perpendicular to the fundus of the internal auditory canal. The anterior (A), posterior (P), superior (S), and inferior (I) aspects of the canals are labeled for ease of orientation. The facial (white arrow), superior vestibular (black arrow), inferior vestibular (black arrowhead), and the cochlear nerves (white arrowhead) are depicted. (b) Right Ear agenesis of the cochlear nerve. (c) Left Ear agenesis of the cochlear nerve. Note on the sagittal oblique section the facial nerve in the anterosuperior quadrant (white arrow). Only one vestibular nerve is seen in the posterior quadrant (black arrowhead), showing incomplete separation of the superior and inferior vestibular nerves. The cochlear nerve is absent in the anterior inferior quadrant (white arrowhead).

Using a three-dimensional (3D) constructive interference in steady state (CISS) MRI sequences, the vestibulocochlear and facial nerves can be thoroughly evaluated. Both nerves have similar cisternal and canalicular courses. They both emerge from the lateral aspect of the lower border of the pons and traverse the cerebellopontine angle cistern at an oblique angle. Next, the nerves cross the inner auditory canal (IAC) in its entire length [4]. Within the internal auditory canal, the vestibulocochlear nerve splits into three parts (cochlear, superior vestibular, and inferior vestibular). These three vestibulocochlear nerve branches, along with the facial nerve, have a characteristic appearance on sagittal oblique 3D CISS cross-sectional images (Figure 1).

The knowledge is limited about etiology of congenital deafness. The radiologic images have become an important diagnostic tool in this regard. The purpose of this paper is to present a case of a 4-year-old boy congenitally deaf who was found to have bilateral agenesis of the cochlea nerve during preoperative workup for cochlear implantation.

## 2. Case Report

 A 4-year-old boy congenitally deaf came from another state for evaluation at the Division of Cochlear Implantation of Brasilia University Medical School Hospital. He had the diagnosis of profound deafness since he was 5 months old, and he started using an external hearing device since then without any practical benefit.

He was born by cesarean section with normal gestation age and normal birth weight. There was no history of familial deafness, and there was no consanguinity between the parents. He had no additional congenital malformations, and he had normal neuropsychomotor development except for the profound bilateral deafness. Adenotonsillectomy and bilateral ventilation tubes placement had been performed in the past.

Audiometry disclosed profound bilateral deafness. The use of the external device improved performance in 500 Hz (15 db in the left ear and 20 db in the right) only. Transient and distortion product evoked otoacoustic emissions were absent on both ears. Brainstem-evoked auditory potentials were also absent on both sides.

**Figure 2:**
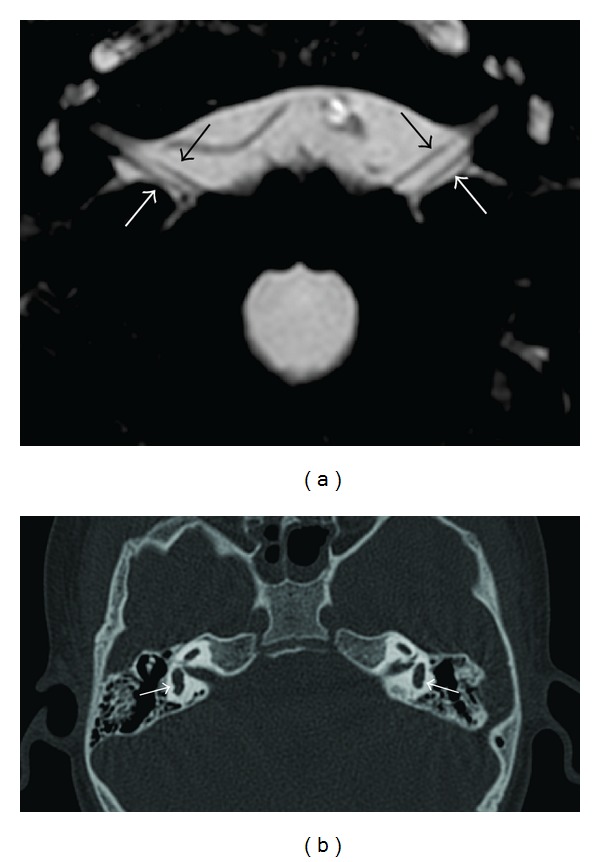
(a) Axial cross-sectional MRI 3D CISS sequence with mean intensity projection through the IAC and cerebellopontine angle. Only two nerves are detected: the facial nerve (black arrow) and the vestibular nerve (white arrow). (b) Axial CT image through the mastoid shows vestibules with dysplastic morphology (white arrow). Note the absence of semicircular canals.

**Figure 3:**
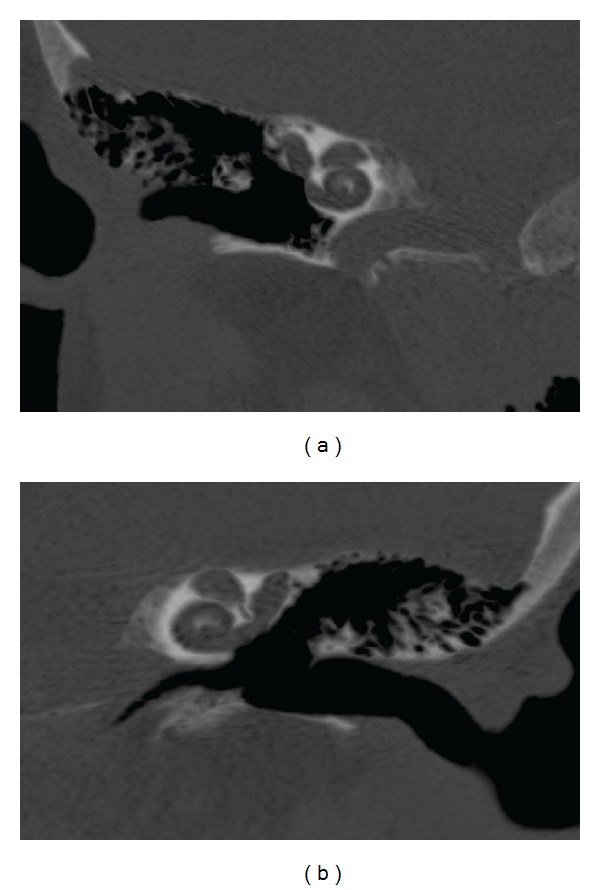
Right (a) and left (b) coronal oblique reformation of the mastoid, using minimal intensity projection. Note the dysplastic morphology of the vestibule and the absence of semicircular canals. The cochlea has normal appearance.

The MRI revealed only two nerves in each IAC, one in the anterior superior quadrant, identified as the facial nerve, and one on the posterior quadrants, representing both the superior and inferior vestibular nerves (Figures 2(a) and 2(b)). The semicircular canals were not seen and the vestibule had dysplastic morphology (Figures 3(a) and 3(b)). The diagnosis was bilateral agenesis of the cochlear nerves and semicircular canals.

## 3. Discussion

 Agenesis of the auditory nerve is estimated to be responsible for 1% of congenital deafness cases [5]. It is characterized by the absence of the auditory nerve in the IAC. Its diagnosis is of extreme importance in preoperative evaluation of a patient who is candidate for cochlear implant. The width of the internal auditory canal and the thickness of the acusticofacial nerve have implications for surgical programming. As the CT has some limitations of definition of neuroanatomical structures, the analysis of the membranous labyrinth and the internal auditory canal must be performed by MRI [6]. MRI cross-sectional images in the sagittal oblique plane are ideal for the detection of cochlear nerve aplasia, hypoplasia, or agenesis because they allow the identification of the facial, cochlear, and vestibular nerves. The aplasia of the cochlear nerve is seen by the absence of the nerve in the anterior superior quadrant. The vestibular nerves may be identified separately or as one only nerve. The latter case is considered an anatomic variant (Figures 1 and 2) [7].

Shelton et al. [8] report a probable association between IAC thickness on CT with the hipoplasia/agenesia of acusticofacial nerve. However, recent studies show that normal IAC and labyrinth can be present in the agenesis of CN. Thereby, IAC thickness is not a landmark trustworthy to indicate the integrity of the CN [2, 5].

A radiological classification of the hypoplasia and aplasia of the vestibulocochlear nerve (VCN) is suggested. It is based on the affected branch and presence of labyrinth dysplasia. Type I: the VCN is affected with presence of IAC stenosis and a dysplastic labyrinth may be present. Type IIa: alteration of the cochlear branch with labyrinth dysplasia (from minor dysplasia to common cavity). Type IIb: normal labyrinth associated to damage of the cochlear branch. Type III: illness of the initial branch of vestibular nerve [9]. The hypoplasia or isolated aplasia of the vestibular branch with presence of the branch to the cochlea has not been described up to now. The present patient was a Type IIb with semicircular canals agenesis and vestibular dysplasia.

There are reports of cochlear implantation (CI) in patients with cochlear malformations and hypoplasia of CN. The performances of these patients are not as good compared to patients treated with a normal anatomy of the cochlea [8, 9]. An auditory brainstem implant is another treatment option for patients with inner ear abnormalities in the presence of cochlear nuclei. In young children, the experience is very limited. This is due to the nature of surgery, as it requires brain surgery to implant the device. The effectiveness of the device is also reduced compared to CI. Recipients only have an awareness of sound and beat. They will not be able to hear melodies. Colletti et al. [10] treated three children with an auditory brainstem implantation device. All three were diagnosed with cochlear nerve aplasia. Six months after the implantation, these patients were able to identify different sounds and distinguish sound frequencies.

## 4. Conclusion

Congenital bilateral cochlear nerve aplasia is a rare entity. The image studies became important tools in the diagnosis of these lesions. CT and MRI can evaluate possible malformations of the labyrinth and vestibulocochlear nerve. In the presence of aplasia of the CN, the cochlear implant is formally contraindicated. Studies with brainstern implantation have showed promising results.
